# Global gene expression reveals stress-responsive genes in *Aspergillus fumigatus* mycelia

**DOI:** 10.1186/s12864-017-4316-z

**Published:** 2017-12-04

**Authors:** Hiroki Takahashi, Yoko Kusuya, Daisuke Hagiwara, Azusa Takahashi-Nakaguchi, Kanae Sakai, Tohru Gonoi

**Affiliations:** 1 Medical Mycology Research Center, Chiba University, 1-8-1 Inohana, Chuo-ku, Chiba 260-8673 Japan; 2 Molecular Chirality Research Center, Chiba University, 1-33 Yayoi-cho, Inage-ku, Chiba 263-8522 Japan

**Keywords:** Transcriptome, RNA-seq, *Aspergillus fumigatus*, Environmental stress, ESR

## Abstract

**Background:**

*Aspergillus fumigatus* is a human fungal pathogen that causes aspergillosis in immunocompromised hosts. *A. fumigatus* is believed to be exposed to diverse environmental stresses in the host cells. The adaptation mechanisms are critical for infections in human bodies. Transcriptional networks in response to diverse environmental challenges remain to be elucidated. To gain insights into the adaptation to environmental stresses in *A. fumigatus* mycelia, we conducted time series transcriptome analyses.

**Results:**

With the aid of RNA-seq, we explored the global gene expression profiles of mycelia in *A. fumigatus* upon exposure to diverse environmental changes, including heat, superoxide, and osmotic stresses. From the perspective of global transcriptomes, transient responses to superoxide and osmotic stresses were observed while responses to heat stresses were gradual. We identified the stress-responsive genes for particular stresses, and the 266 genes whose expression levels drastically fluctuated upon exposure to all tested stresses. Among these, the 77 environmental stress response genes are conserved in *S. cerevisiae*, suggesting that these genes might be more general prerequisites for adaptation to environmental stresses. Finally, we revealed the strong correlations among expression profiles of genes related to ‘rRNA processing’.

**Conclusions:**

The time series transcriptome analysis revealed the stress-responsive genes underlying the adaptation mechanisms in *A. fumigatus* mycelia. These results will shed light on the regulatory networks underpinning the adaptation of the filamentous fungi.

**Electronic supplementary material:**

The online version of this article (10.1186/s12864-017-4316-z) contains supplementary material, which is available to authorized users.

## Background

Billions of people are infected with fungi every year in the world [1]. Filamentous fungus *Aspergillus fumigatus* is a ubiquitous fungus commonly found in soil, but is also reported as a major cause of invasive fungal aspergillosis infections in humans, especially in patients with compromised or suppressed host immunity [2–4].

Inhalation of airborne conidia is responsible for human infection [3, 4]. When *A. fumigatus* establishes an infection in the human lung, the mycelia must respond to highly variable conditions, which might impose stress on the fungal pathogen [4, 5]. Thus, the adaptation mechanism in mycelia plays an important role in terms of pathogenicity of *A. fumigatus*. Among environmental conditions, tolerance to higher temperatures is a critical trait for mammalian infections [6]. It is conceivable that oxidative stress needs to be overcome, as reactive oxygen species produced by host cells, such as neutrophils, damage the fungal pathogen [7–9]. Osmotic stress adaptation and sensitivity are also supposed to be important for conidial germination, growth and virulence [10–14]. It has been reported that MAP kinase SakA plays a pivotal role in responses and adaptation to osmotic stress [10].

Transcriptome analyses are particularly useful for obtaining a deeper understanding of gene regulation in organisms from bacteria to mammals. Microarray technology has been applied to *A. fumigatus* since whole genome sequencing was initiated in 2005 [6]. Nierman et al. (2005) have conducted time series microarray analyses upon exposure to heat shock of both 37 °C and 48 °C from 30 °C [6]. Do et al. (2009) have addressed the transcriptional networks by state space models using microarray data for more than 2000 genes studied by Nierman et al. (2005), and revealed the expression profiles of heat shock proteins, such as *hsp70* and *hsp30*, in response to heat stress [15]. Putative targets of *hsf1* were identified by integrative analysis of proteome and transcriptome [16].

In yeast, genome-wide gene expression analyses under various changes in the extracellular environment have been investigated by microarray [17–19]. Notably, Gasch et al. (2000) have proposed the environmental stress response (ESR) genes that responded to almost all the stressful conditions [18]. The ESR is a general adaptive response to suboptimal environments [18]. Indeed, Emri et al. (2015) identified 116 ESR genes in response to five different oxidative stress conditions in *A. nidulans* [20]. Some genes of *A. fumigatus* related to heat stress are orthologs of genes of *S. cerevisiae* [6], indicating that the stress response in *A. fumigatus* might be partially related to the response of the ESR genes.

It is noted that RNA-seq technology appears to be the most powerful tool for transcriptome analysis, and has great potential to investigate the transcriptome in *A. fumigatus*, e.g. developmental stages [21–27]. It is demonstrated that RNA-seq data were better correlated with proteome data than microarray data in *A. fumigatus* [24]. So far, no studies have investigated the integrative stress responses in *A. fumigatus*, such as seeking the ESR genes. In the present study, we explored the global transcriptome responses by RNA-seq in *A. fumigatus* mycelia upon exposure to heat, superoxide, and osmotic stresses.

## Results

### Time series transcriptome analyses in response to diverse environmental stresses

To determine the genes that respond to several stress conditions, we performed time series transcriptome analyses of mycelia of *A. fumigatus* Af293 using RNA-seq under four environmental conditions: heat stress from 30 °C to 37 °C (hereafter, HS1), heat stress from 30 °C to 48 °C (HS2), superoxide stress by adding menadione (SS), and osmotic stress by adding sorbitol (OS). Following each stress, RNA samples from the mycelia were harvested at six time points: 0, 15, 30, 60, 120, and 180 min (Table 1). A total of 277,417,888 sequence reads were obtained by a MiSeq for HS1, and a HiSeq for HS2, SS, and OS (Additional file 1: Table S1, Additional file 2: Table S2).

**Table 1:** Experimental conditions

Condition	Medium	Sampling point
Heat stress from 30 °C to 37 °C (HS1)	YG	0 (unshocked), 15, 30, 60, 120, and 180 min
Heat stress from 30 °C to 48 °C (HS2)		
Superoxide stress by menadione (SS)	AMM	
Osmotic stress by sorbitol (OS)		

FPKM values of 9840 genes were calculated as described in the Methods (Additional file 3: Table S3). To evaluate the expression levels in unshocked mycelia (0 min), we calculated Pearson correlation coefficients using 6932 genes that showed FPKM values higher than the median value in at least one condition (Additional file 4: Figure S1). There were high correlations between 0 min data of HS1, HS2, SS, and OS, e.g. *r* = 0.81 for HS1 and HS2, and *r* = 0.98 for SS and OS. Next, to elucidate transcriptome changes in the treated mycelia, the log2-transformed ratio values to the expression levels in unshocked mycelia were calculated, and used throughout this study. To illustrate global trends of transcriptome in response to HS1, HS2, SS, and OS, we performed principal component analysis (PCA) using 6932 genes (Additional file 5: Figure S2). The transcriptome of HS2 appeared to significantly differ from other three stresses. We observed similar behavior in the transcriptome of SS and OS. In addition, it appeared that the gene expression in response to SS and OS was transiently changed.

**Fig. 1:**
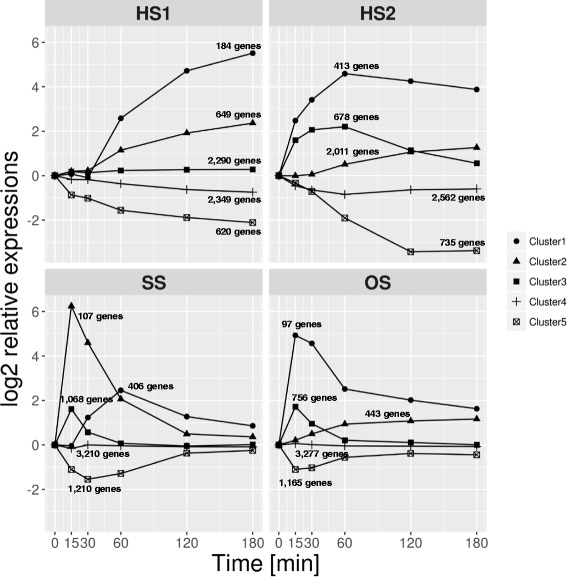
K-means clustering of transcription profiles using five centroids. The average log2-transformed ratio values were plotted. Among 6092 genes in HS1, clusters 1, 2, 3, 4, and 5 consist of 184 (3.0%), 649 (10.7%), 2290 (37.6%), 2349 (38.6%), and 620 (10.2%) genes, respectively. Among 6399 genes in HS2, clusters 1, 2, 3, 4, and 5 consist of 413 (6.5%), 2011 (31.4%), 678 (10.6%), 2562 (40.0%), and 735 (11.5%) genes. Among 6001 genes in SS, clusters 1, 2, 3, 4, and 5 consist of 406 (6.8%), 107 (1.8%), 1068 (17.8%), 3210 (53.5%), and 1210 (20.2%) genes. Among 5738 genes in OS, clusters 1, 2, 3, 4, and 5 consist of 97 (1.7%), 443 (7.7%), 756 (13.2%), 3277 (57.1%), and 1165 (20.3%) genes

Furthermore, we conducted K-means clustering analysis (Fig. 1). The relatively low expressed genes were excluded, and the genes with FPKM values higher than the median value of at least one time point for each stress were used: 6092 genes in HS1, 6399 in HS2, 6001 in SS, and 5738 in OS. The expression of 184 genes enriched in the ‘oxidation-reduction process’ was gradually up-regulated throughout HS1, and maximized at 180 min (Fig. 1, cluster 1). That of the 620 genes enriched in ‘translation’ was down-regulated (cluster 5). Meanwhile, the expression of the 413 (cluster 1) enriched in ‘rRNA processing’ gradually increased throughout HS2, and that of the 678 genes (cluster 3) enriched in ‘transmembrane transport’ gradually increased until 60 min upon exposure to HS2. The 735 genes (cluster 5) enriched in the ‘oxidation-reduction process’ decreased during exposure to HS2.

In contrast to heat stresses, the transient dynamics in response to SS and OS were observed. The expression of the 107 genes (cluster 2) enriched in the ‘oxidation-reduction process’ largely increased in SS at 15 min, and then reverted back to a similar level for the unshocked condition. The expression levels of catalase genes, *cat1* (Afu3g02270) and *cat2* (Afu8g01670), in cluster 2 were 30.1- and 241.8-fold up-regulated at 15 min. The 1210 genes (cluster 5) enriched in ‘translation’ transiently decreased at 30 min. Similarly, the 97 genes (cluster 1) transiently responded to OS. The genes related to the high-osmolarity glycerol response (HOG) pathway, e.g. *dprA* (Afu4g00860), *dprB* (Afu6g12180), *dprC* (Afu7g04520), *ptcD* (Afu5g13740), and *atfD* (Afu6g12150), were observed in cluster 1 [12, 28–30]. Consistent with previous reports, the expression levels of *dprA* and *dprB* were 99.4- and 71.3-fold up-regulated at 15 min [12]. In addition, we observed that the expression of MAPK *sakA* (Afu1g12940) in cluster 2 as a major component of HOG pathway was 6.6-fold up-regulated. Taken together, two types of adaptation in response to suboptimal conditions were observed, namely gradual shift in HS1 and HS2, and transient shift in SS and OS.

### Identification of the stress-responsive genes in response to environmental changes

**Fig. 2:**
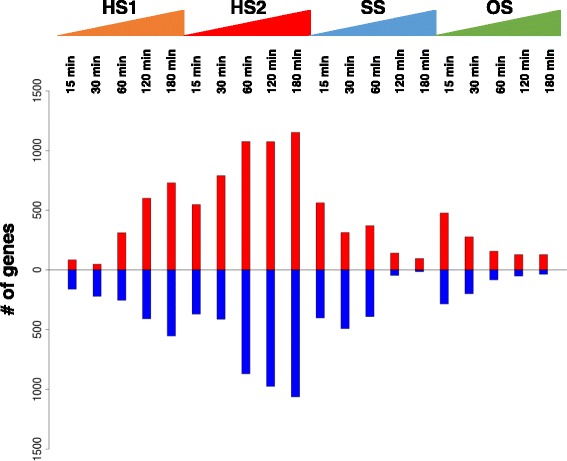
Bar plots of the numbers of genes with altered expression at each time point. Red and blue indicate the numbers of up- and down-regulated genes, respectively

We sought stress-responsive genes and identified 1598, 3383, 1735, and 1085 genes as the stress-responsive genes whose expression levels were differentially expressed in HS1, HS2, SS, and OS, respectively (Fig. 2). The thresholds used for judging whether genes responded to stress were >3-fold or <1/3-fold. In HS1, the maximum numbers of differentially expressed genes were measured at 180 min; that is, 730 up- and 553 down-regulated genes. Likewise, in HS2, 1152 and 1063 as up- and down-regulated genes, respectively, were measured at 180 min. In SS, 564 up- and 491 down-regulated genes were detected at 15 and 30 min, respectively. Furthermore, in OS, 478 up- and 284 down-regulated genes were observed at 15 min. Consistent with the results by K-means analysis and PCA (Fig. 1, Additional file 5: Figure S2), the gene expression upon exposure to heat stresses was gradually changed, while transient changes were observed in SS and OS.

### Comparison with microarray data of heat stress

**Fig. 3:**
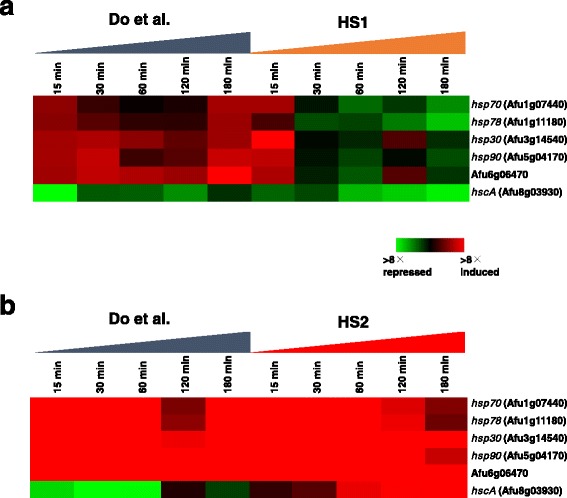
Comparison of the gene expression of six heat shock proteins between microarray and RNA-seq data. **a** Expression profiles of six heat shock proteins in response to HS1. **b** Expression profiles of six heat shock proteins in response to HS2. Color scale is indicated

We identified 1598 (HS1) and 3383 (HS2) genes as described above. Do et al. (2009) have reported 726 and 2200 genes for heat shock of 37 °C and 48 °C based on microarray data, respectively [6, 15]. Comparing identified genes, 268 and 1044 genes were observed in both experiments of 37 °C and 48 °C, respectively, and almost all the expression profiles of these genes appear to be consistent (Additional file 6: Figure S3). According to Do et al. (2009), six heat shock genes were identified as the hub nodes by network analysis, namely *hsp70* (Afu1g07440), *hsp78* (Afu1g11180), *hsp30* (Afu3g14540), *hsp90* (Afu5g04170), Afu6g06470, and *hscA* (Afu8g03930). In response to heat shock of 37 °C, the expression levels of *hsp78*, *hsp30*, *hsp90*, and Afu6g06470 were up-regulated at 15 min in our HS1 data, although those were up-regulated throughout the time series in Do et al. (2009) (Fig. 3a). The expression profile of *hscA* was consistent across both experiments. In response to heat shock of 48 °C, the expression profiles except for *hscA* were similar, while that of *hscA* exhibited the opposite trend (Fig. 3b).

### Acute responses upon exposure to superoxide and osmotic stresses

**Fig. 4:**
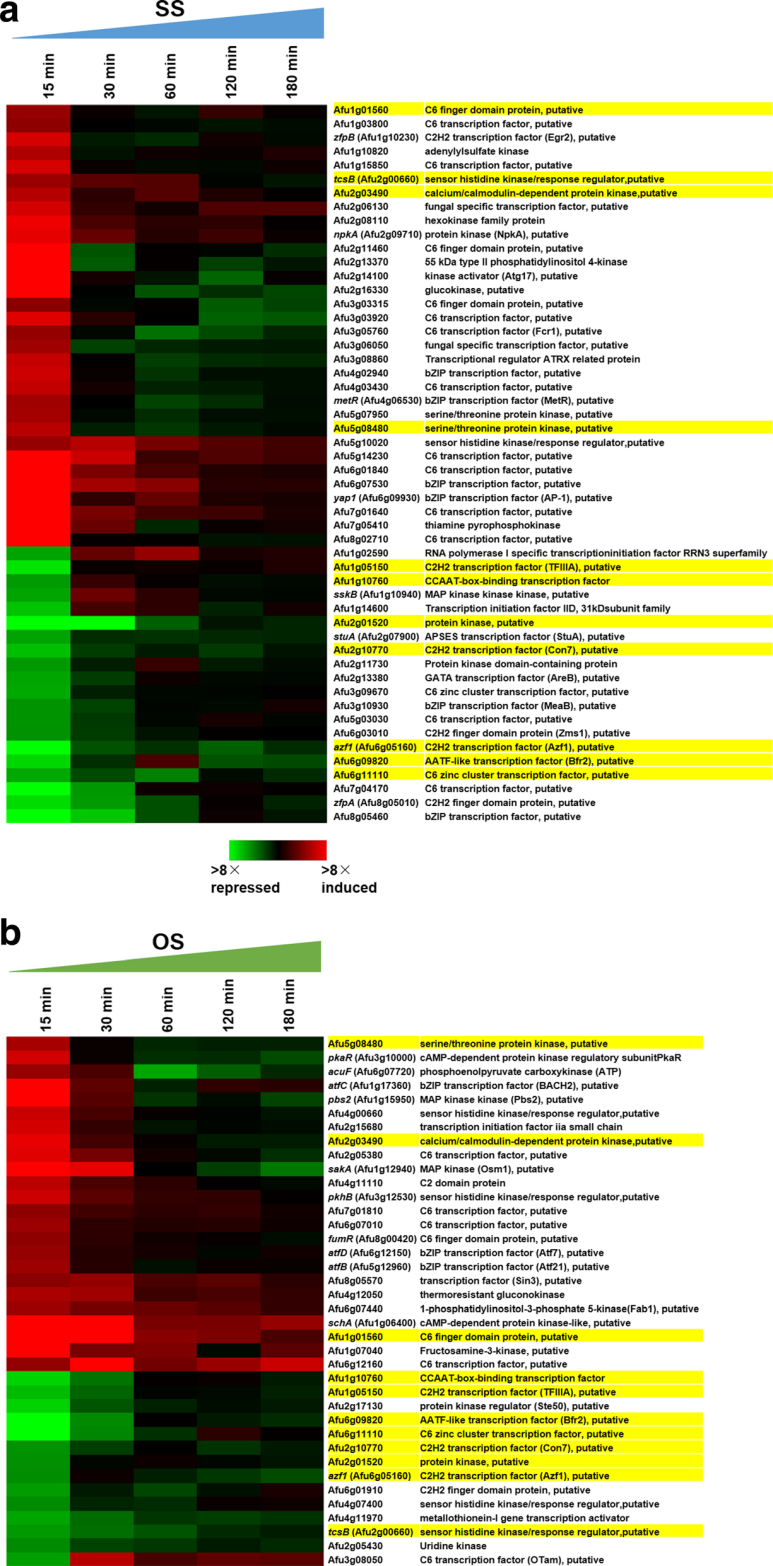
Heat map of regulator genes upon exposure to SS and OS. **a** The expression profiles of 46 regulator genes. Thirty-two and 14 genes were up- and down-regulated at 15 min in SS, respectively. **b** The expression profiles of 44 regulator genes. Twenty-four and 20 genes were up- and down-regulated at 15 min in OS, respectively. The yellow boxes indicate 11 genes with altered expression at 15 min under both SS and OS conditions

Among the stress-responsive genes, 892 (51%) in SS and 728 (67%) in OS genes were up/down-regulated at one or both of 15 and 30 min, while 141 (9%) in HS1 and 332 (10%) in HS2 were observed. This indicated that the majority of the genes respond to SS and OS in a rapid and transient manner. As kinase and transcription factor (TF) play an important role in sensing superoxide and osmotic stresses [15, 31–33], we sought such regulator genes from the set of genes that responded to SS and OS. A total of 32 and 24 regulator genes, including kinase and transcriptional factor, were up-regulated at 15 min in SS and OS, respectively, while 14 and 20 genes were down-regulated at 15 min (Fig. 4a, b). Consistent with the observation that TF *yap1* (Afu6g09930) responded to H_2_O_2_ [9], we observed that the expression of *yap1* was 5.7-fold up-regulated at 15 min in response to SS. The expression of histidine kinase *phkB* (Afu3g12530) was detected as transiently responsive genes in OS. The expression of *sakA* was not changed in response to SS, although it was up-regulated in response to OS, concordant with a previous study [34]. The expression of MAPKKK *sskB* (Afu1g10940) was 5.3-fold changed at 15 min. Interestingly, the expression of histidine kinase *tcsB* was transiently up-regulated at 15 min in response to HS1 and SS, while it was down-regulated at 15 min in response to OS. This is consistent with the report that *tcsB* is negatively regulated by osmotic stress in *A. nidulans* and *A. fumigatus* [31, 35]. The expression of *tcsB* was up-regulated at 15, 30, and 60 min in response to HS2. In addition, we observed the up-regulation of Atf family genes including *atfB* (Afu5g12960), *atfC* (Afu1g17360), and *atfD* under the OS condition, while the expression of *atfA* (Afu3g11330) was 2.6-fold changed at 15 min [28, 35]. Comparing the genes observed in SS and OS, three (Afu1g01560, Afu2g03490, and Afu5g08480) and seven (Afu1g05150, Afu1g10760, Afu2g01520, Afu2g10770, *azf1* (Afu6g05160), Afu6g09820, and Afu6g11110) uncharacterized genes were up- and down-regulated at 15 min under both SS and OS conditions, respectively.

### Characterization of the stress-responsive genes

**Fig. 5:**
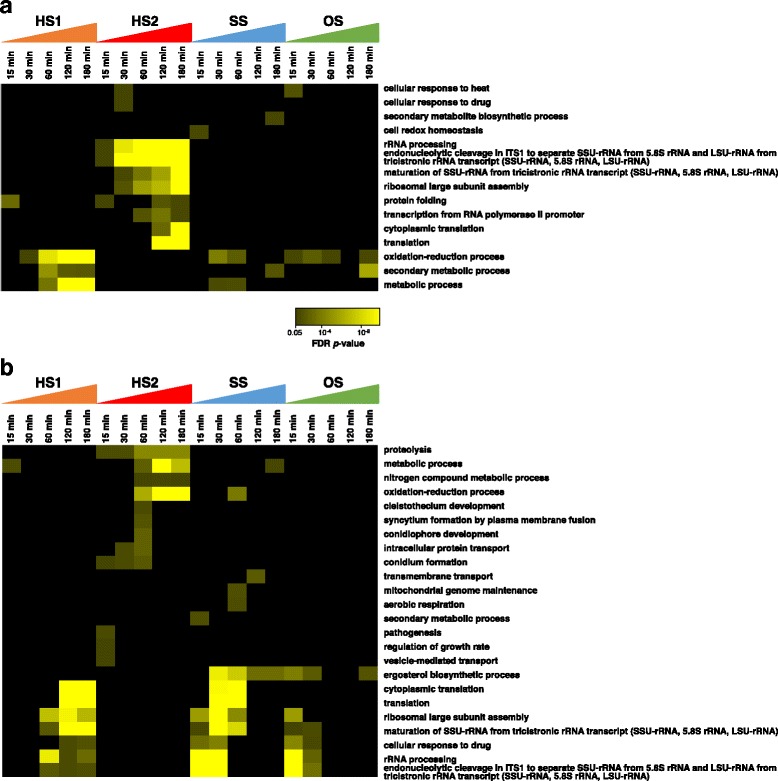
Heat map of overrepresented GO terms based on FDR *p*-value. **a** 15 GO terms for up-regulated genes. **b** 24 GO terms for down-regulated genes. Color scale is indicated

**Fig. 6:**
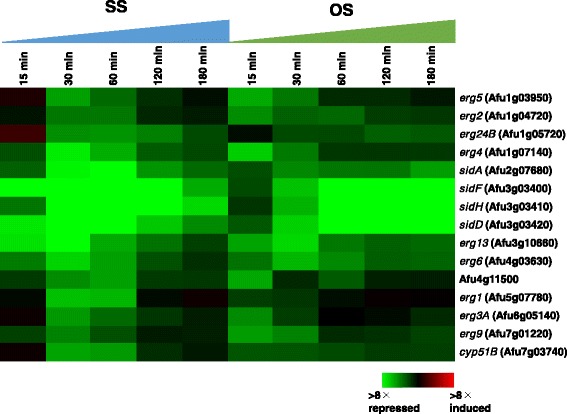
Expression profiles of 15 genes annotated as ‘ergosterol biosynthetic process’ in response to SS and OS. Color scale is indicated

Next, to investigate which gene functions are associated with the individual stresses, Gene Ontology (GO) enrichment analysis was conducted. In total, 56, 67, and 89 GO terms for the categories Cellular Component, Molecular Function, and Biological Process, respectively, were tested. Among 89 GO terms associated with Biological Process, 15 GO terms were overrepresented for up-regulated genes (Fig. 5a), while 24 GO terms were overrepresented for down-regulated genes (Fig. 5b). In HS2, GO terms related to ribosome, such as ‘rRNA processing’, ‘endonucleolytic cleavage in ITS1 to separate SSU-rRNA from 5.8S rRNA and LSU-rRNA from tricistronic rRNA transcript (SSU-rRNA, 5.8S rRNA, LSU-rRNA)’, ‘maturation of SSU-rRNA from tricistronic rRNA transcript (SSU-rRNA, 5.8S rRNA, LSU-rRNA)’, and ‘ribosomal large subunit assembly’, were up-regulated throughout the time series. The expression of genes annotated as ‘protein folding’ was up-regulated at 15 min in both HS1 and HS2. In SS, the expression of genes annotated as ‘cell redox homeostasis’ was up-regulated at 15 min. A total of 203 genes annotated as ‘oxidation-reduction process’ were up-regulated upon exposure to HS1, SS, and OS. Among them, 15 genes were up-regulated under all HS1, SS, and OS conditions, indicating that these are essential for oxidation reduction in *A. fumigatus*. Intriguingly, ‘ergosterol biosynthetic process’ was overrepresented in response to SS and OS (Figs. 5b, 6). The expression levels of 15 genes annotated as ‘ergosterol biosynthetic process’, including *erg6*, *erg2*, *erg3A*, *erg5*, and *erg4*, were down-regulated under one or both of the SS and OS conditions (Fig. 6).

### Identification of the stress-responsive genes upon exposure to all tested stresses

**Fig. 7:**
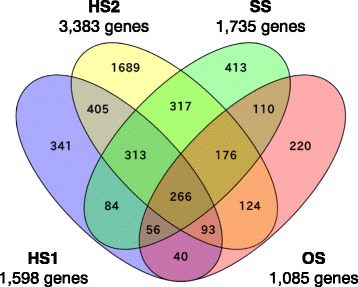
Venn diagram of the stress-responsive genes in HS1 (1598 genes), HS2 (3383 genes), SS (1735 genes), and OS (1085 genes). A total of 266 genes responded to all four conditions. This figure was drawn using Venny 2.0 [36]

**Fig. 8:**
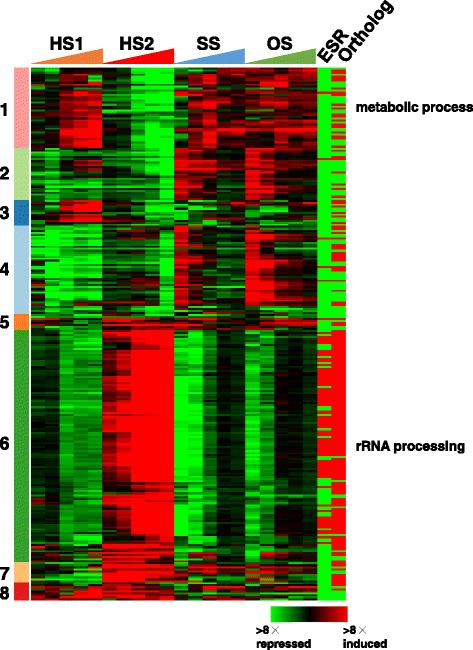
Heat map of the 266 stress-responsive genes. Color scale is indicated. ESR and Ortholog indicate the existence of ESR and orthologous genes in *S. cerevisiae*, respectively. Red and green represent true and false, respectively, unlike the color scale of gene expression. The ‘cutree’ function in R was used to divide 266 genes into eight clusters. Clusters 1, 2, 3, 4, 5, 6, 7, and 8 consist of 40 (15.0%), 26 (9.8%), 13 (4.9%), 44 (16.5%), 8 (3.0%), 116 (43.6%), 10 (3.8%), and 9 (3.4%) genes, respectively

We sought the genes with altered expression under all four conditions (Fig. 7) [36]. Among 4647 genes, 266 were differentially expressed upon exposure to all four tested conditions. These genes were classified into eight clusters (Fig. 8, Additional file 7: Table S4). According to the functional annotations of genes in AspGD, 29 and 50 genes were annotated with ‘conserved hypothetical protein’ and ‘hypothetical protein’, respectively. Notably, the expression levels of 116 genes related to ‘rRNA processing’ were up-regulated in response to HS2, while they were transiently down-regulated in response to SS and OS (cluster 6). The expression levels of 40 genes related to ‘metabolic process’ were up-regulated under HS1, SS, and OS conditions, while they were down-regulated under the HS2 condition (cluster 1). Furthermore, we observed nine and 14 genes whose expression levels were up-regulated and down-regulated under all four conditions, respectively. The expression of Afu5g09910 encoding a putative *p*-nitroreductase-family protein was drastically activated upon exposure to all tested conditions. In particular, the expression was 709-fold changed at 15 min in SS. The expression of *msdS* (Afu1g14560) was transiently down-regulated upon exposure to HS1, SS, and OS. Especially, the expression was decreased to 1/22-fold at 120 min under the HS2 condition. *msdS* encodes a class I 1,2-α-mannosidase, and the cell wall integrity of the *msdS* mutant was slightly affected at a higher temperature [37]. In addition, five TFs, Afu1g04140 (cluster 1), Afu8g00420 (*fumR*) (cluster 1), Afu1g03800 (cluster 4), Afu3g11170 (cluster 4), and Afu6g12160 (cluster 7), were identified as stress-responsive genes.

### Comparison with yeast ESR genes

In both *S. cerevisiae* and *Schizosaccharomyces pombe*, the ESR genes that respond to diverse types of environmental stresses have been identified [17, 18]. Particularly, in *S. cerevisiae*, Gasch et al. (2000) proposed 867 ESR genes including different functions, e.g. energy generation and storage, defense against reactive oxygen species, and DNA repair [18]. Here we attempted to identify *A. fumigatus* genes that are orthologous to the *S. cerevisiae* ESR (ScESR) genes. First, we conducted ortholog identification by reciprocal best-hit pairs against *S. cerevisiae* proteins, and consequently, 768 (768/867 ScESR genes, 88.6%) genes in *A. fumigatus* were identified as orthologs of ScESR genes, while among 9840 genes in *A. fumigatus*, 5399 (54.9%) genes corresponded to the genes of *S. cerevisiae*. Notably, the expression of 229 genes (29.8%) of the 768 *A. fumigatus* genes was not observed under any stress conditions tested. Among the 768 *A. fumigatus* genes, 77 (10.0%) were the stress-responsive genes identified above (Additional file 7: Table S4). As shown in Fig. 8, 71 up-regulated genes in HS2 were orthologous to ScESR genes, such as chaperones and ribosomal processing protein (Fig. 8, cluster 6). The genes in *A. fumigatus* identified as the ScESR ortholog were significantly overrepresented in 266 stress-responsive genes (Fisher’s exact test, *p*-value 2.35E − 25).

### Correlation analysis reveals the clusters of co-expressed genes

**Fig. 9:**
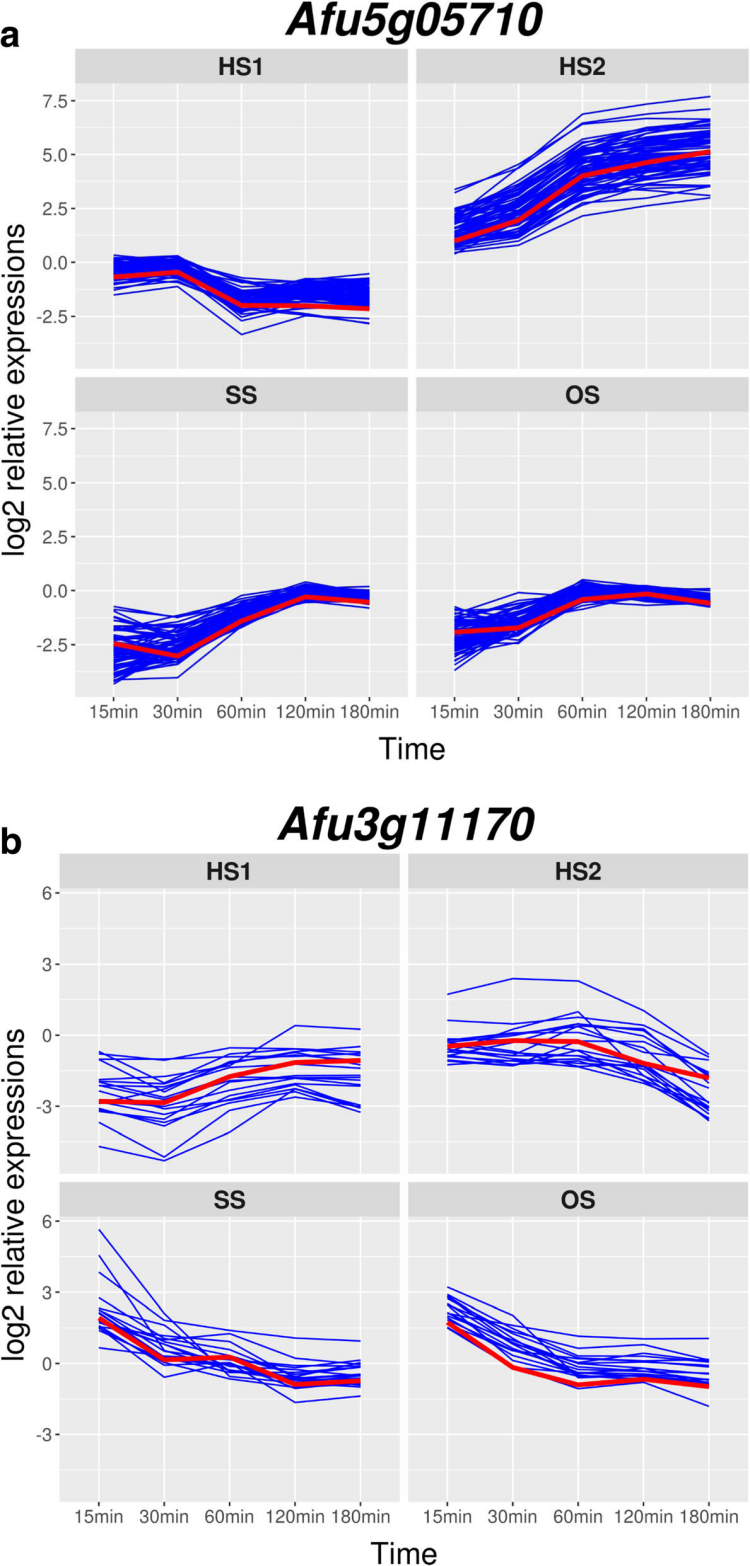
Expression profiles of co-expressed genes. **a** Expression profiles of Afu5g05710 (red line) and 73 genes. **b** Expression profiles of Afu3g11170 (red line) and 18 genes. x-axis and y-axis correspond to the time after shocking the mycelia, 15, 30, 60, 120 and 180 min, and log2-transformed ratio values, respectively

Finally, we explored the co-expressed genes through four stress conditions. Co-expression relationships are useful for unravelling the transcriptional networks, such as TF-regulated genes [38–42]. By using normalized data, i.e. 4490 genes, correlation analyses were conducted. Gene-to-gene Pearson correlation coefficients were calculated, and the Gap statistic as described in the Methods was applied to estimate the cluster size [43]. We surveyed the hub gene, and observed Afu5g05710 annotated as pseudouridylate synthase family protein, whose expression correlated with 233 genes with *r* > 0.95. Seventy-three genes (*r* > 0.98) were estimated as co-expressed genes, 44 of which (cluster 6) were stress-responsive genes (Fig. 9a). These expression levels were up-regulated in HS2, while they were down-regulated in HS1, SS, and OS. The expression profiles of genes related to ‘rRNA processing’ were highly correlated to those of Afu5g05710 in response to all tested stresses. Furthermore, 18 genes were estimated as co-expressed genes with uncharacterized TF Afu3g11170 (*r* > 0.87) (Fig. 9b). These genes transiently responded to SS and OS at 15 min, while they were down-regulated upon exposure to HS1 and HS2.

## Discussion

The aim of this study is to comprehensively elucidate the stress-responsive genes under diverse environmental stress conditions in pathogenic filamentous fungi *A. fumigatus*. We conducted time series transcriptome analyses by RNA-seq upon exposure to four different environmental stresses: heat stress from 30 °C to 37 °C (HS1) and from 30 °C to 48 °C (HS2), superoxide stress by adding menadione (SS), and osmotic stress by adding sorbitol (OS). The 0 min data were highly correlated (Additional file 4: Figure S1). We concluded that our RNA-seq data would be consistent in the initial growth stage, and the differences of growth time could have little impact. Furthermore, even though additional AMM was added in OS, the correlation between 180 min data of SS and OS was quite high (*r* = 0.95), and 180 min (T5) data of OS was positioned closely to that of SS (Additional file 5: Figure S2), indicating that the data of SS and OS could be compatible.

### Comparison with microarray data

When compared with the previously published microarray data [15], we found that the expression profiles of heat shock genes were similar (Fig. 3). The profile of one of the heat shock genes, *hscA*, was different between two data sets, while the profiles of the other five genes were consistent. In addition, we comprehensively compared our HS1 and HS2 expression data with the microarray data (Additional file 6: Figure S3). The correlations between them for HS1 (37 °C) (*n* = 676 × 5 time points) and HS2 (48 °C) were 0.30 (*p*-value, 1.22E − 70) and 0.39 (*p*-value, <1.00E − 70), respectively. It has been reported that the expression profile correlation between microarray and RNA-seq in *A. fumigatus* ranges from 0.14 to 0.41 [24]. Thus, we concluded that our RNA-seq data are comparable with previous microarray data. We newly identified 1275 and 2254 genes that were up- or down-regulated in HS1 and HS2, respectively. It will be necessary to investigate the characterization of those genes in future. In our RNA-seq data, 36 and 94 genes that had been identified in microarray were missed because there were no mapping sequences in HS1 and HS2, respectively. This is most likely because these genes were misidentified by microarray based on the fluorescence intensity.

### Stress-specific genes

We comprehensively revealed how the genes responded to specific stress conditions. A total of 39 GO terms were identified (Fig. 5a, b). In HS1, the expression of 117 genes annotated as ‘oxidation-reduction process’ was up-regulated, consistent with the observation that the genes involved in balancing the redox state were up-regulated upon heat stress treatment [16]. Although ‘oxidation-reduction process’ was not statistically overrepresented in HS2, the expression of 67 genes annotated as ‘oxidation-reduction process’ was up-regulated, indicating that the heat stress to some extent triggered the redox imbalance. Probably, enhancement of oxygen respiration by heat shock may cause the redox imbalance as previously reported in yeast [44]. It has been reported that the oxidative stress response leads to mitochondrial dysfunction in yeast [45] and *A. fumigatus* [9]. Consistent with these observations, the expression of five genes (Afu1g07450, Afu2g04270, Afu3g14490, Afu5g03640, and Afu5g07140) annotated as ‘mitochondrial genome maintenance’ was down-regulated at 60 min in SS (Fig. 5b). Interestingly, the expression of Afu3g14490, ketol-acid reductoisomerase, was differentially expressed upon exposure to airway epithelial cells in conidia [46], suggesting that Afu3g14490 may play important roles for environmental changes, e.g. oxidative damage, in both hyphae and conidia.

Upon heat stress, Hsf1 induces the expression of many genes, including chaperones as one of the key regulators in fungi including yeast and *A. fumigatus* [16]. In our transcriptome data, the expression of *hsf1* was largely induced in response to HS2 until 60 min, and slightly induced at 15 min in response to OS (2.5-fold). The expression levels of two genes, i.e. uncharacterized protein Afu4g10360 and MAP kinase kinase *pbs2* (Afu1g15950), were strongly correlated with those of *hsf1* (*r* > 0.95). The yeast ortholog of Afu4g10360, ETP1, is required for growth on ethanol and ethanol-induced transcriptional activation [47], suggesting that Afu4g10360 may sense environmental changes such as carbon source, heat stress, and osmotic stress, and relate to the transcriptional regulations. Although the growth defect of *Δpbs2* strain was not observed at 48 °C [32], *Δpbs2* strain was hypersensitive to oxidative and osmotic stresses in *A. fumigatus* [13], indicating that the acute induction of *pbs2* may be a prerequisite for adaptation to oxidative and osmotic stresses.

The expression of the genes involved in ‘ergosterol biosynthetic process’ was down-regulated under SS and OS conditions (Figs. 5b, 6). This is consistent with the fact that repression of ergosterol biosynthesis is essential for stress resistance and important for salt stress in yeast [48]. Accordingly, the down-regulation of ‘ergosterol biosynthetic process’ might be prerequisite for adaptation to environmental challenges in *A. fumigatus* hyphae in terms of cell wall integrity. The expression of *srbB* (Afu4g03460) was 1/5-fold down-regulated at 30 min in SS. *srbB* is involved in the regulation of ergosterol biosynthesis upon hypoxia [49], suggesting that *srbB* would respond to not only hypoxia but also oxidative stress. Yap1 is known to function against H_2_O_2_ and menadione [9]. Consistent with the previous report, in our transcriptome analyses, *yap1* was identified as an SS-specific gene (Fig. 4a). The expression of *yap1* was up-regulated 5.7-fold at 15 min in response to SS, but did not change under other conditions (Additional file 8: Figure S4). Interestingly, the expression levels of some of the putative Yap1 target genes identified by Lessing et al. (2007) [9] were differentially expressed in not only SS but also HS2 and OS. For example, the expression of *cat2* responded to SS, HS2, and OS. The expression of *aspf3* (Afu6g02280) was induced in response to HS1, HS2, and SS, consistent with up-regulation upon exposure to heat stress [16]. This suggested that *cat2* and *aspf3* could be regulated not only by Yap1 but also other TFs. Among 28 putative Yap1 target genes, 14 genes, such as *aof2* (Afu4g13000), were not detected as SS-responsive genes.

### The stress-responsive genes in *A. fumigatus*

We identified the 266 stress-responsive genes that were differentially expressed upon exposure to all four tested environmental changes, indicating that these genes are essential for mycelia of filamentous fungi to sense and respond to the environmental changes. We observed that most of these genes are involved in ribosome-related functions. The set of stress-responsive genes included 171 orthologs and 95 non-orthologs to *S. cerevisiae*. The proportion of *S. cerevisiae* ortholog genes was largely similar for the whole genome (54.9%) and the stress-responsive genes (64.3%). Among the 171 ortholog genes, 77 (45.0%) are orthologous to ScESR genes, which is relatively high compared with the genome-wide orthologs between *A. fumgiatus* and *S. cerevisiae* (768 orthologs to ScESR for 5399 ortholog genes, 14.2%). This suggested that the responses of ESR genes would be more frequently conserved between *S. cerevisiae* and *A. fumigatus.* On the contrary, *A. fumigatus* stress-responsive genes included 94 genes (35.3%) that are orthologous to *S. cerevisiae* non-ESR genes. These genes might have evolved to respond to and sense the environmental changes in filamentous fungi, namely by transcriptional evolution. The remainder of the 95 genes (35.7%) have no ortholog in *S. cerevisiae*, suggesting that these genes have uniquely evolved in *A. fumigatus*, likely along with the spreading of its habitat.

Notably, the Afu5g09910 encoding a putative *p*-nitroreductase-family protein was drastically induced in response to all tested conditions. Its protein abundance is regulated by Yap1 against H_2_O_2_ [9]. In addition, it has been reported that the protein abundance and gene expression of Afu5g09910 are up-regulated upon exposure to human neutrophils and gliotoxin, respectively [50, 51]. This suggested that the expression of Afu5g09910 may be induced by diverse environmental challenges.

### Correlation analysis

Finally, we conducted correlation analysis to estimate the co-expressed genes with a particular gene. The expression of Afu5g05710 was highly correlated with 73 genes enriched in ‘rRNA processing’. Particularly, it was strongly induced under the HS2 condition, suggesting that these genes might be a prerequisite for protecting against sub-lethal exposures. The regulation of those genes, however, remains unknown.

## Conclusion

Global gene expression analysis under diverse environmental conditions is important for understanding the pathogenicity of the opportunistic pathogen *A. fumigatus*. Here we performed time series transcriptome analysis under four different conditions, and identified stress-responsive genes for particular stresses. In addition, we identified the 266 genes whose expression levels were drastically changed upon exposure to heat, superoxide, and osmotic stresses in *A. fumigatus*. The 77 ESR genes common in filamentous fungi *A. fumigatus* and unicellular yeast *S. cerevisiae* responded to diverse environmental stresses, suggesting that these genes might be more general prerequisites for adaptation to environmental stresses. Further investigation of the molecular mechanisms will shed light on the adaptation in response to suboptimal conditions.

## Methods

### Fungal strain and growth condition

The strain used in this study was *A. fumigatus* Af293. Potato dextrose agar (PDA; Difco, Detroit, USA), YG [27], and AMM [52] were regularly used for culturing the strain. To collect fresh conidia, the stored conidia were incubated on a PDA plate at 30 °C, or an AMM at 37 °C  for 1 week.

### Environmental stress

#### Heat stress from 30 °C to 37 °C or 48 °C (HS1 or HS2)

Mycelia were grown in YG medium at 30 °C for 17 h before being transferred to a water bath of 37 °C or 48 °C. The samples in individual culture flasks were collected at 0, 15, 30, 60, 120, and 180 min after the transfer, and harvested mycelium was frozen in liquid nitrogen.

#### Superoxide stress (SS)

Mycelia were grown in AMM medium at 37 °C for 24 h. Following superoxide stress by adding menadione (Sigma-Aldrich, St. Louis, USA) for a final concentration of 10 μM menadione, the samples were collected at 0, 15, 30, 60, 120, and 180 min, and harvested mycelium was frozen in liquid nitrogen.

#### Osmotic stress (OS)

Mycelia were grown in AMM medium 37 °C for 24 h. Following the addition of AMM supplemented with 2 M sorbitol (Wako, Osaka, Japan) for a final concentration of 1 M sorbitol, the samples were collected at 0, 15, 30, 60, 120, and 180 min, and harvested mycelium was frozen in liquid nitrogen.

### RNA extraction

After frozen mycelia were disrupted with zirconia beads using a multi-beads shocker (Yasui Kikai, Osaka, Japan), total RNAs were extracted using an RNeasy Mini Kit (Qiagen, Hilden, Germany) and contaminating genomic DNAs were removed with an RNase-Free DNase set (Qiagen, Hilden, Germany) according to the manufacturer’s instructions.

### RNA sequencing

TruSeq RNA Sample Prep Kit v2 (Illumina, San Diego, USA) and KAPA Stranded mRNA-Seq Kit (Kapa Biosystems, Wilmington, USA) were employed to prepare the libraries of mRNA samples for multiplexed sequencing according to the manufacturers’ protocol (Additional file 1: Table S1). The qualities of all libraries were determined by an Agilent 2100 Bioanalyzer (Agilent Technologies, Santa Clara, USA). Paired-end 25 bp and single-end 60 bp were performed with the aid of a MiSeq (Illumina) and a HiSeq1500 (Illumina), respectively.

### Expression analysis

Illumina data sets were trimmed using Trimmomatic (ver. 0.33), where sequencing adapters and sequences with low-quality scores were removed [53]. Cleaned reads were mapped to the genome sequence of *A. fumigatus* Af293 (29,420,142 bp; genome version: s03-m05-r04) from AspGD [54] using STAR (ver. 2.4.2a) with the default parameters other than ‘--alignIntronMax 1000’ [55]. FPKMs were calculated using cuffdiff in Cufflinks (ver. 2.2.1) with default parameters [56]. Data analyses were conducted using the R programming language (https://www.r-project.org/), corrplot (ver. 0.84) [57], ggplot2 (ver. 1.0.1) [58] and cummerbund (ver. 2.18.0) software [59].

### Identification of yeast orthologous genes

The amino acid sequences of 6713 proteins (downloaded on 13th January 2016) in *S. cerevisiae* were obtained from the Saccharomyces Genome Database (SGD, http://www.yeastgenome.org). A list of 867 ESR genes was obtained from Gasch et al. (2000) [18]. The reciprocal best-hit pairs between *A. fumigatus* and *S. cerevisiae* by BLASTP (ver. 2.2.28+) [60] analysis with ‘-evalue 1e-4’ were used to identify the ortholog genes.

### GO analysis

Genes were functionally categorized using their GO information [61] obtained from AspGD, and overrepresented GO terms were identified using Fisher’s exact test. The one-tailed Fisher’s exact *p*-value corresponding to the overrepresentation of GO categories with equal to or greater than 20 genes was calculated based on counts in 2 × 2 contingency tables. *p*-values were corrected by the false discovery rate method [62], and the threshold was set as 0.05.

### Estimation of the cluster size using the gap statistic

Pearson correlation coefficient was applied to identify the co-expressed genes targeted by particular genes, e.g. TFs. To estimate cluster size, we adopted the Gap statistic proposed by Hastie et al. (2000) [43]. We assumed that gene expression levels in a cluster with *k* genes are highly coherent, and measured them by calculating *R* ^2^(*k*).

1 $$ {R}^2(k)=\frac{V_B}{V_T} $$

*V*_*B*_ and *V*_*T*_ are between variance and total variance, respectively. We obtained a randomized data matrix by permuting the elements within each gene expression, and calculated *R* ^2^(*k*)^∗^. We then defined the Gap statistic as the observed *R* ^2^(*k*) minus the mean of *R* ^2^(*k*)^∗^.

2 $$ Gap(k)={R}^2(k)- mean\left({R}^2{(k)}^{\ast}\right) $$

The estimated cluster size $\widehat{k}$ is given by:

3 $$ \widehat{k}={argmax}_k Gap(k) $$

In this study, we generated 1000 permutated matrixes and estimated the cluster size $\widehat{k}$.

## Additional files

Additional file 1: Table S1. Information of RNA-seq. (XLSX 9 kb)

Additional file 2: Table S2. Mapping results. (XLSX 11 kb)

Additional file 3: Table S3. FPKM values of 9840 genes. (XLSX 2563 kb)

Additional file 4: Figure S1. Correlation matrix between 0 min data of HS1, HS2, SS, and OS. Size of the circles is proportional to the correlation coefficient [57]. (PDF 45 kb)

Additional file 5: Figure S2. Principal component analysis (PCA). Size of the circles indicates time after exposure to the stress, i.e. 15 (T1), 30 (T2), 60 (T3), 120 (T4), and 180 (T5) min. (PDF 91 kb)

Additional file 6: Figure S3. Overview of heat stress genes. (a) Comparison of 268 genes identified in Do et al. (2009) and this study for heat stress of 37 °C. (b) Comparison of 1044 genes for heat stress of 48 °C. Color scale is indicated. (PDF 47 kb)

Additional file 7: Table S4. log2 transformed ratio values of the 266 genes. (XLSX 289 kb)

Additional file 8: Figure S4. Expression profiles of 27 putative Yap1 target genes reported by Lessing et al. (2007). *yap1* was up-regulated only at 15 min in response to SS. (PDF 85 kb)

## References

[1] Brown GD, Denning DW, Levitz SM (2012). Tackling human fungal infections. Science.

[2] Thom C, Raper KB (1945). A manual of the Aspergilli.

[3] Dagenais TR, Keller NP (2009). Pathogenesis of *Aspergillus fumigatus* in invasive Aspergillosis. Clin Microbiol Rev.

[4] Kousha M, Tadi R, Soubani AO (2011). Pulmonary aspergillosis: a clinical review. Eur Respir Rev.

[5] Brown AJ, Haynes K, Quinn J (2009). Nitrosative and oxidative stress responses in fungal pathogenicity. Curr Opin Microbiol.

[6] Nierman WC, Pain A, Anderson MJ, Wortman JR, Kim HS, Arroyo J, Berriman M, Abe K, Archer DB, Bermejo C (2005). Genomic sequence of the pathogenic and allergenic filamentous fungus *Aspergillus fumigatus*. Nature.

[7] Chauhan N, Latge JP, Calderone R (2006). Signalling and oxidant adaptation in *Candida albicans* and *Aspergillus fumigatus*. Nat Rev Microbiol.

[8] Philippe B, Ibrahim-Granet O, Prévost MC, Gougerot-Pocidalo MA, Sanchez Perez M, Van der Meeren A, Latgé JP (2003). Killing of *Aspergillus fumigatus* by alveolar macrophages is mediated by reactive oxidant intermediates. Infect Immun.

[9] Lessing F, Kniemeyer O, Wozniok I, Loeffler J, Kurzai O, Haertl A, Brakhage AA (2007). The *Aspergillus fumigatus* transcriptional regulator AfYap1 represents the major regulator for defense against reactive oxygen intermediates but is dispensable for pathogenicity in an intranasal mouse infection model. Eukaryot Cell.

[10] Xue T, Nguyen CK, Romans A, May GS (2004). A mitogen-activated protein kinase that senses nitrogen regulates conidial germination and growth in *Aspergillus fumigatus*. Eukaryot Cell.

[11] Brown NA, Goldman GH (2016). The contribution of *Aspergillus fumigatus* stress responses to virulence and antifungal resistance. J Microbiol.

[12] Winkelströter LK, Bom VL, de Castro PA, Ramalho LN, Goldman MH, Brown NA, Rajendran R, Ramage G, Bovier E, Dos Reis TF (2015). High osmolarity glycerol response PtcB phosphatase is important for *Aspergillus fumigatus* virulence. Mol Microbiol.

[13] Ma D, Li R (2013). Current understanding of HOG-MAPK pathway in *Aspergillus fumigatus*. Mycopathologia.

[14] de Castro PA, Chiaratto J, Winkelströter LK, Bom VL, Ramalho LN, Goldman MH, Brown NA, Goldman GH (2014). The involvement of the Mid1/Cch1/Yvc1 calcium channels in *Aspergillus fumigatus* virulence. PLoS One.

[15] Do JH, Yamaguchi R, Miyano S (2009). Exploring temporal transcription regulation structure of *Aspergillus fumigatus* in heat shock by state space model. BMC Genomics.

[16] Albrecht D, Guthke R, Brakhage AA, Kniemeyer O (2010). Integrative analysis of the heat shock response in *Aspergillus fumigatus*. BMC Genomics.

[17] Causton HC, Ren B, Koh SS, Harbison CT, Kanin E, Jennings EG, Lee TI, True HL, Lander ES, Young RA (2001). Remodeling of yeast genome expression in response to environmental changes. Mol Biol Cell.

[18] Gasch AP, Spellman PT, Kao CM, Carmel-Harel O, Eisen MB, Storz G, Botstein D, Brown PO (2000). Genomic expression programs in the response of yeast cells to environmental changes. Mol Biol Cell.

[19] Strassburg K, Walther D, Takahashi H, Kanaya S, Kopka J (2010). Dynamic transcriptional and metabolic responses in yeast adapting to temperature stress. OMICS.

[20] Emri T, Szarvas V, Orosz E, Antal K, Park H, Han KH, JH Y, Pócsi I (2015). Core oxidative stress response in *Aspergillus nidulans*. BMC Genomics.

[21] Gibbons JG, Beauvais A, Beau R, McGary KL, Latgé JP, Rokas A (2012). Global transcriptome changes underlying colony growth in the opportunistic human pathogen *Aspergillus fumigatus*. Eukaryot Cell.

[22] Hagiwara D, Takahashi H, Kusuya Y, Kawamoto S, Kamei K, Gonoi T (2016). Comparative transcriptome analysis revealing dormant conidia and germination associated genes in *Aspergillus* species: an essential role for AtfA in conidial dormancy. BMC Genomics.

[23] McDonagh A, Fedorova ND, Crabtree J, Yu Y, Kim S, Chen D, Loss O, Cairns T, Goldman G, Armstrong-James D (2008). Sub-telomere directed gene expression during initiation of invasive aspergillosis. PLoS Pathog.

[24] Müller S, Baldin C, Groth M, Guthke R, Kniemeyer O, Brakhage AA, Valiante V (2012). Comparison of transcriptome technologies in the pathogenic fungus *Aspergillus fumigatus* reveals novel insights into the genome and MpkA dependent gene expression. BMC Genomics.

[25] O'Keeffe G, Hammel S, Owens RA, Keane TM, Fitzpatrick DA, Jones GW, Doyle S (2014). RNA-seq reveals the pan-transcriptomic impact of attenuating the gliotoxin self-protection mechanism in *Aspergillus fumigatus*. BMC Genomics.

[26] Rokas A, Gibbons JG, Zhou X, Beauvais A, Latgé JP (2012). The diverse applications of RNA-seq for functional genomic studies in *Aspergillus fumigatus*. Ann N Y Acad Sci.

[27] Takahashi-Nakaguchi A, Muraosa Y, Hagiwara D, Sakai K, Toyotome T, Watanabe A, Kawamoto S, Kamei K, Gonoi T, Takahashi H (2015). Genome sequence comparison of *Aspergillus fumigatus* strains isolated from patients with pulmonary aspergilloma and chronic necrotizing pulmonary aspergillosis. Med Mycol.

[28] Hagiwara D, Asano Y, Marui J, Yoshimi A, Mizuno T, Abe K (2009). Transcriptional profiling for *Aspergillus nidulans* HogA MAPK signaling pathway in response to fludioxonil and osmotic stress. Fungal Genet Biol.

[29] Hagiwara D, Suzuki S, Kamei K, Gonoi T, Kawamoto S (2014). The role of AtfA and HOG MAPK pathway in stress tolerance in conidia of *Aspergillus fumigatus*. Fungal Genet Biol.

[30] Hagiwara D, Sakamoto K, Abe K, Gomi K (2016). Signaling pathways for stress responses and adaptation in *Aspergillus* species: stress biology in the post-genomic era. Biosci Biotechnol Biochem.

[31] Miskei M, Karányi Z, Pócsi I (2009). Annotation of stress-response proteins in the aspergilli. Fungal Genet Biol.

[32] Ji Y, Yang F, Ma D, Zhang J, Wan Z, Liu W, Li R (2012). HOG-MAPK signaling regulates the adaptive responses of *Aspergillus fumigatus* to thermal stress and other related stress. Mycopathologia.

[33] Hagiwara D, Takahashi-Nakaguchi A, Toyotome T, Yoshimi A, Abe K, Kamei K, Gonoi T, Kawamoto S (2013). NikA/TcsC histidine kinase is involved in conidiation, hyphal morphology, and responses to osmotic stress and antifungal chemicals in *Aspergillus fumigatus*. PLoS One.

[34] Reyes G, Romans A, Nguyen CK, May GS (2006). Novel mitogen-activated protein kinase MpkC of *Aspergillus fumigatus* is required for utilization of polyalcohol sugars. Eukaryot Cell.

[35] Pereira Silva L, Alves de Castro P, Dos Reis TF, Paziani MH, Von Zeska Kress MR, Riaño-Pachón DM, Hagiwara D, Ries LN, Brown NA, Goldman GH (2017). Genome-wide transcriptome analysis of *Aspergillus fumigatus* exposed to osmotic stress reveals regulators of osmotic and cell wall stresses that are SakA(HOG1) and MpkC dependent. Cell Microbiol.

[36] Oliveros JC. Venny. An interactive tool for comparing lists with Venn's diagrams. 2007–2015. http://bioinfogp.cnb.csic.es/tools/venny/index.html

[37] Li Y, Zhang L, Wang D, Zhou H, Ouyang H, Ming J, Jin C (2008). Deletion of the msdS/AfmsdC gene induces abnormal polarity and septation in *Aspergillus fumigatus*. Microbiology.

[38] Harbison CT, Gordon DB, Lee TI, Rinaldi NJ, Macisaac KD, Danford TW, Hannett NM, Tagne JB, Reynolds DB, Yoo J (2004). Transcriptional regulatory code of a eukaryotic genome. Nature.

[39] Kobayashi H, Akitomi J, Fujii N, Kobayashi K, Altaf-Ul-Amin M, Kurokawa K, Ogasawara N, Kanaya S (2007). The entire organization of transcription units on the *Bacillus subtilis* genome. BMC Genomics.

[40] Redestig H, Weicht D, Selbig J, Hannah MA (2007). Transcription factor target prediction using multiple short expression time series from *Arabidopsis thaliana*. BMC Bioinformatics.

[41] Takahashi H, Morioka R, Ito R, Oshima T, Altaf-Ul-Amin M, Ogasawara N, Kanaya S (2011). Dynamics of time-lagged gene-to-metabolite networks of *Escherichia coli* elucidated by integrative omics approach. OMICS.

[42] Wada M, Takahashi H, Altaf-Ul-Amin M, Nakamura K, Hirai MY, Ohta D, Kanaya S (2012). Prediction of operon-like gene clusters in the *Arabidopsis thaliana* genome based on co-expression analysis of neighboring genes. Gene.

[43] Hastie T, Tibshirani R, Eisen MB, Alizadeh A, Levy R, Staudt L, Chan WC, Botstein D, Brown P (2000). ‘Gene shaving’ as a method for identifying distinct sets of genes with similar expression patterns. Genome Biol.

[44] Sugiyama K, Izawa S, Inoue Y (2000). The Yap1p-dependent induction of glutathione synthesis in heat shock response of *Saccharomyces cerevisiae*. J Biol Chem.

[45] Demasi AP, Pereira GA, Netto LE (2006). Yeast oxidative stress response. Influences of cytosolic thioredoxin peroxidase I and of the mitochondrial functional state. FEBS J.

[46] Oosthuizen JL, Gomez P, Ruan J, Hackett TL, Moore MM, Knight DA, Tebbutt SJ. Dual organism transcriptomics of airway epithelial cells interacting with conidia of *Aspergillus fumigatus*. PLoS One. 2011;6:e20527. 10.1371/journal.pone.0020527 PMC3105077 21655222

[47] Snowdon C, Schierholtz R, Poliszczuk P, Hughes S, van der Merwe G (2009). ETP1/YHL010c is a novel gene needed for the adaptation of *Saccharomyces cerevisiae* to ethanol. FEMS Yeast Res.

[48] Montañés FM, Pascual-Ahuir A, Proft M (2011). Repression of ergosterol biosynthesis is essential for stress resistance and is mediated by the Hog1 MAP kinase and the Mot3 and Rox1 transcription factors. Mol Microbiol.

[49] Chung D, Barker BM, Carey CC, Merriman B, Werner ER, Lechner BE, Dhingra S, Cheng C, Xu W, Blosser SJ (2014). ChIP-seq and in vivo transcriptome analyses of the *Aspergillus fumigatus* SREBP SrbA reveals a new regulator of the fungal hypoxia response and virulence. PLoS Pathog.

[50] Sugui JA, Kim HS, Zarember KA, Chang YC, Gallin JI, Nierman WC, Kwon-Chung KJ (2008). Genes differentially expressed in conidia and hyphae of *Aspergillus fumigatus* upon exposure to human neutrophils. PLoS One.

[51] Carberry S, Molloy E, Hammel S, O'Keeffe G, Jones GW, Kavanagh K, Doyle S (2012). Gliotoxin effects on fungal growth: mechanisms and exploitation. Fungal Genet Biol.

[52] Kusuya Y, Hagiwara D, Sakai K, Yaguchi T, Gonoi T, Takahashi H (2017). Transcription factor Afmac1 controls copper import machinery in *Aspergillus fumigatus*. Curr Genet.

[53] Bolger AM, Lohse M, Usadel B (2014). Trimmomatic: a flexible trimmer for Illumina sequence data. Bioinformatics.

[54] Cerqueira GC, Arnaud MB, Inglis DO, Skrzypek MS, Binkley G, Simison M, Miyasato SR, Binkley J, Orvis J, Shah P (2014). The *Aspergillus* genome database: multispecies curation and incorporation of RNA-Seq data to improve structural gene annotations. Nucleic Acids Res.

[55] Dobin A, Davis CA, Schlesinger F, Drenkow J, Zaleski C, Jha S, Batut P, Chaisson M, Gingeras TR. STAR: ultrafast universal RNA-seq aligner. Bioinformatics. 2013;29:15–21. 10.1093/bioinformatics/bts635 PMC3530905 23104886

[56] Trapnell C, Williams BA, Pertea G, Mortazavi A, Kwan G, van Baren MJ, Salzberg SL, Wold BJ, Pachter L (2010). Transcript assembly and quantification by RNA-Seq reveals unannotated transcripts and isoform switching during cell differentiation. Nat Biotechnol.

[57] Wei T, Simko V. R package “corrplot”: visualization of a correlation matrix (Version 0.84). 2017. https://github.com/taiyun/corrplot.

[58] Wickham H (2009). ggplot2: elegant graphics for data analysis.

[59] Goff L, Trapnell C, Kelley D. cummeRbund: analysis, exploration, manipulation, and visualization of cufflinks high-throughput sequencing data. R package version 2.18.0. 2013.

[60] Altschul SF, Madden TL, Schäffer AA, Zhang J, Zhang Z, Miller W, Lipman DJ. Gapped BLAST and PSI-BLAST: a new generation of protein database search programs. Nucleic Acids Res. 1997;25:3389–402. 10.1093/nar/25.17.3389 PMC146917 9254694

[61] Ashburner M, Ball CA, Blake JA, Botstein D, Butler H, Cherry JM, Davis AP, Dolinski K, Dwight SS, Eppig JT (2000). Gene ontology: tool for the unification of biology. The gene ontology consortium. Nat Genet.

[62] Benjamini Y, Hochberg Y (1995). Controlling the false discovery rate: a practical and powerful approach to multiple testing. J R Statist Soc B.

