# Supplementary figures and images for: Global gene expression reveals stress-responsive genes in Aspergillus fumigatus mycelia
Source: BMC Genomics. 2017 Dec 4;18:942. doi: 10.1186/s12864-017-4316-z (PMC5715996; doi:10.1186/s12864-017-4316-z)

Figure S1

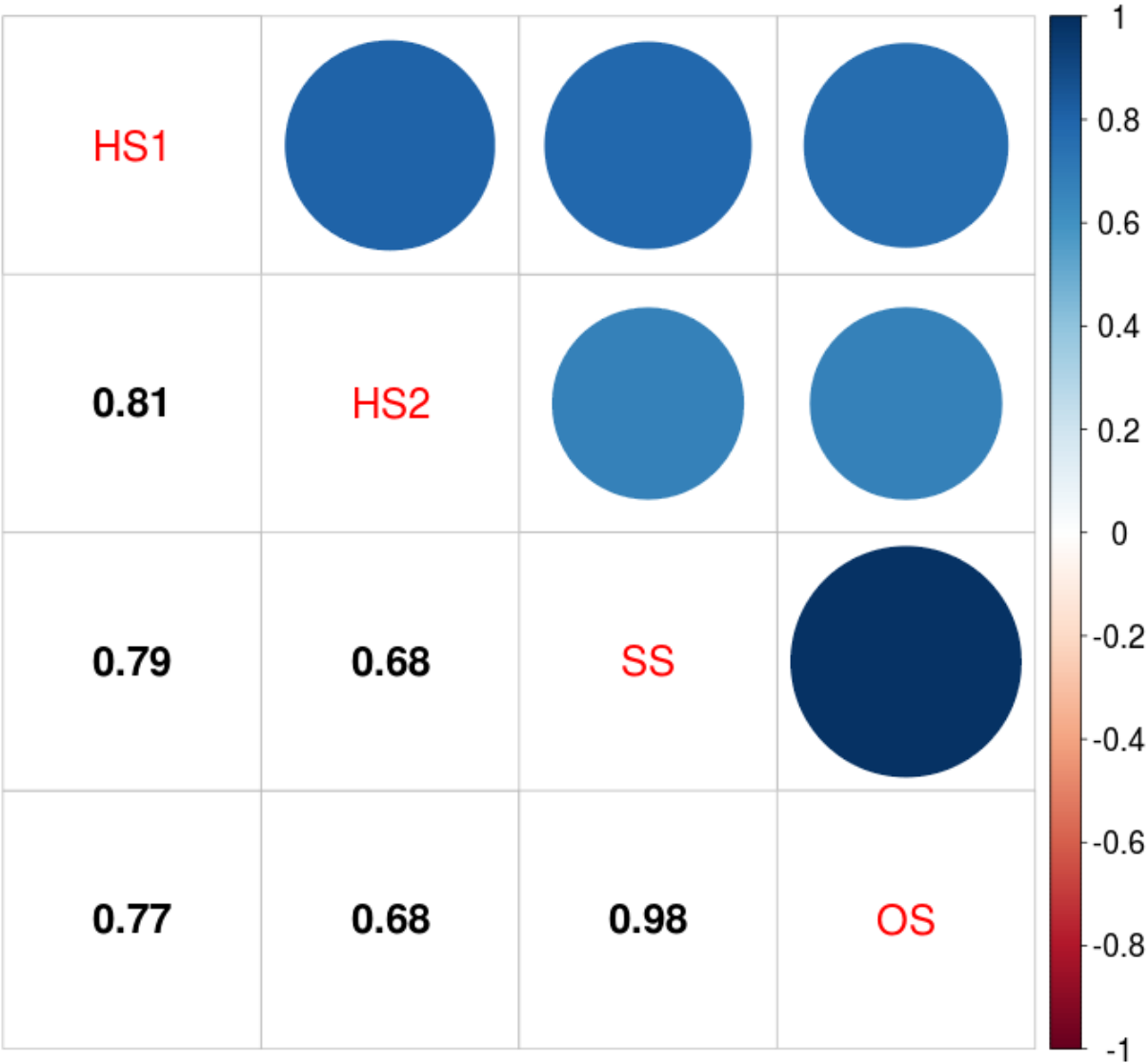

Supplement: Supplementary file 4 — Correlation matrix between 0 min data of HS1, HS2, SS, and OS. Size of the circles is proportional to the correlation coefficient [57]. (PDF 45 kb) [file 12864_2017_4316_MOESM4_ESM.pdf]

Figure S2

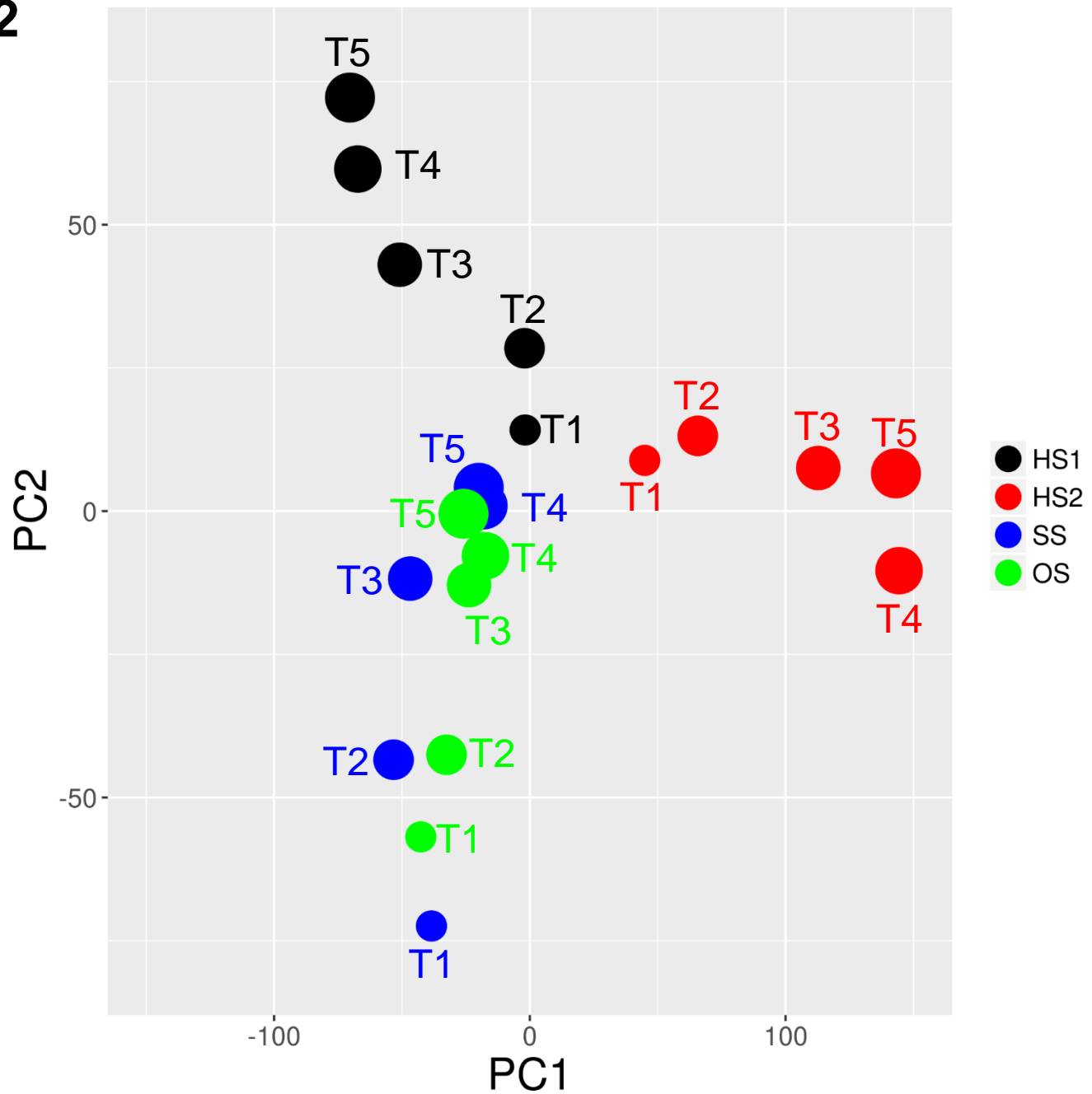

Supplement: Supplementary file 5 — Principal component analysis (PCA). Size of the circles indicates time after exposure to the stress, i.e. 15 (T1), 30 (T2), 60 (T3), 120 (T4), and 180 (T5) min. (PDF 91 kb) [file 12864_2017_4316_MOESM5_ESM.pdf]

Figure S3

a

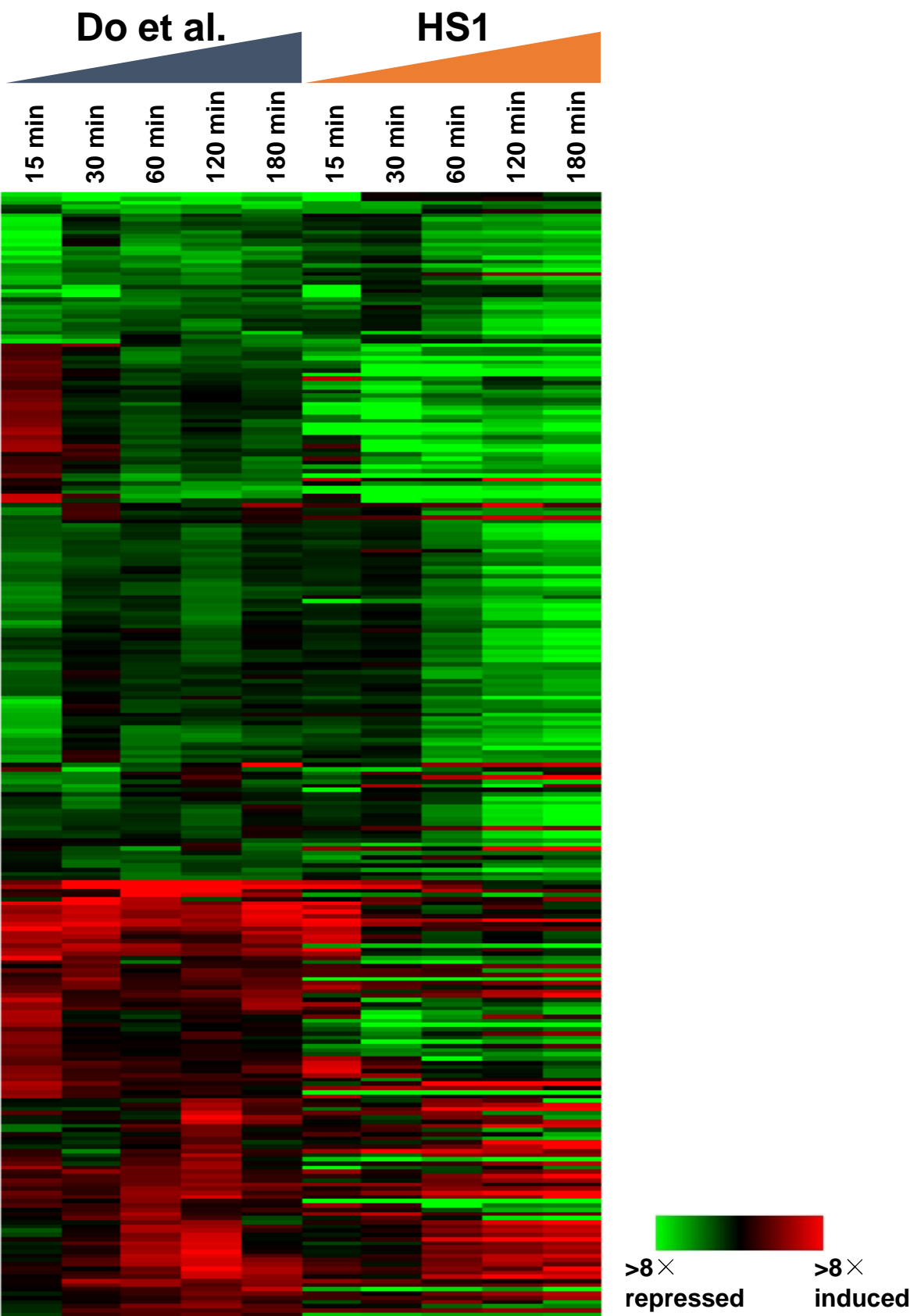

**b**

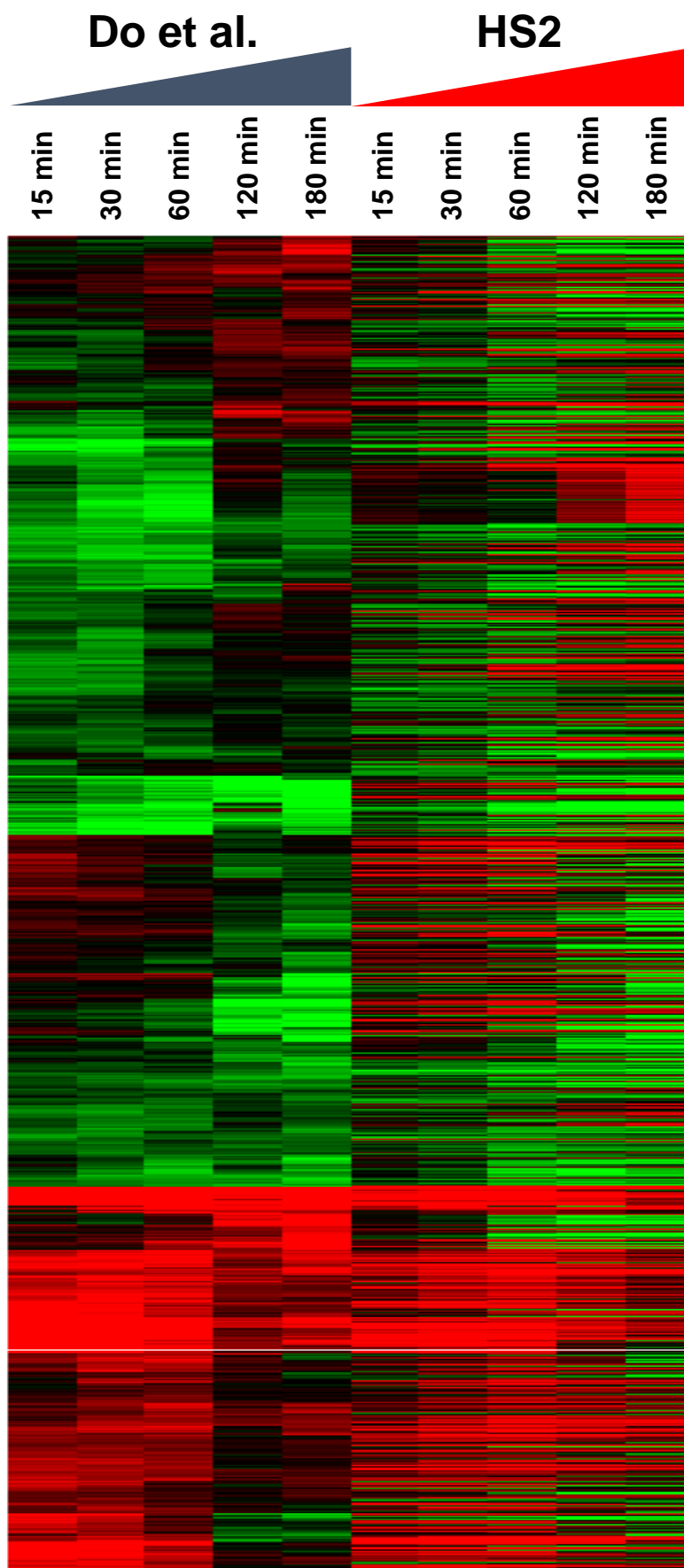

Supplement: Supplementary file 6 — Overview of heat stress genes. (a) Comparison of 268 genes identified in Do et al. (2009) and this study for heat stress of 37 °C. (b) Comparison of 1044 genes for heat stress of 48 °C. Color scale is indicated. (PDF 47 kb) [file 12864_2017_4316_MOESM6_ESM.pdf]

Figure S4    HS1                      HS2                      SS                      OS

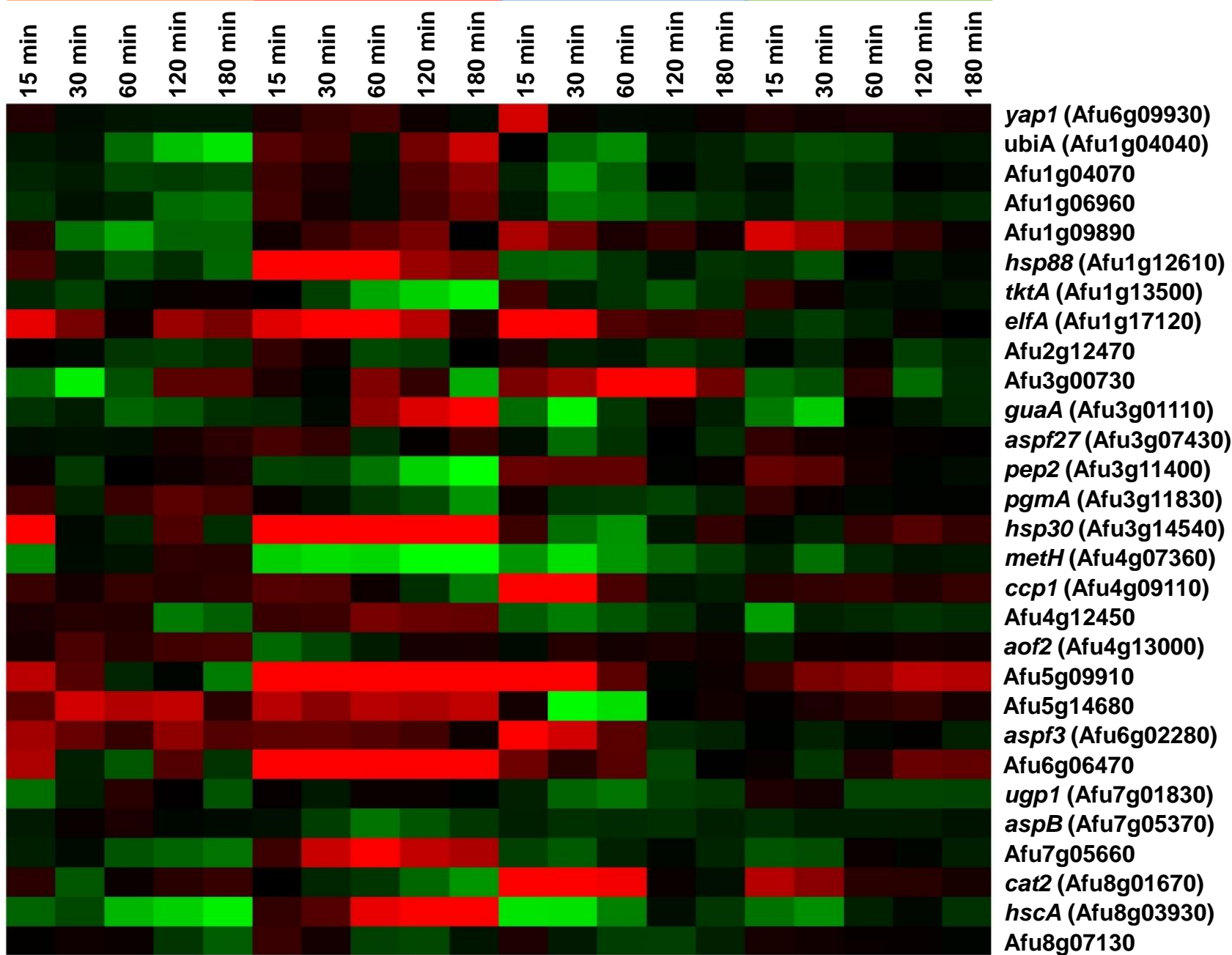

Supplement: Supplementary file 8 — Expression profiles of 27 putative Yap1 target genes reported by Lessing et al. (2007). yap1 was up-regulated only at 15 min in response to SS. (PDF 85 kb) [file 12864_2017_4316_MOESM8_ESM.pdf]
